# Actionable Variants of Unknown Significance in Inherited Arrhythmogenic Syndromes: A Further Step Forward in Genetic Diagnosis

**DOI:** 10.3390/biomedicines12112553

**Published:** 2024-11-08

**Authors:** Estefanía Martínez-Barrios, Andrea Greco, José Cruzalegui, Sergi Cesar, Nuria Díez-Escuté, Patricia Cerralbo, Fredy Chipa, Irene Zschaeck, Miguel Fogaça-da-Mata, Carles Díez-López, Elena Arbelo, Simone Grassi, Antonio Oliva, Rocío Toro, Georgia Sarquella-Brugada, Oscar Campuzano

**Affiliations:** 1 Pediatric Arrhythmias, Inherited Cardiac Diseases and Sudden Death Unit, Hospital Sant Joan de Déu, 08950 Esplugues de Llobregat, Spain; estefania.martinez@sjd.es (E.M.-B.); andrea.greco@sjd.es (A.G.); josecarlos.cruzalegui@sjd.es (J.C.); sergio.cesar@sjd.es (S.C.); nuria.diez@sjd.es (N.D.-E.); patricia.cerralbo@sjd.es (P.C.); fredy.chipa@sjd.es (F.C.); irene.zschaeck@sjd.es (I.Z.); mgmfdamata@gmail.com (M.F.-d.-M.); georgia@brugada.org (G.S.-B.); 2 Pediatric Arrhythmias, Genetic Cardiology and Sudden Death, Cardiovascular Diseases in the Development, Institut de Recerca Sant Joan de Déu, 08950 Esplugues de Llobregat, Spain; 3 European Reference Network for Rare, Low Prevalence and Complex Diseases of the Heart (ERN GUARD-Heart), 1105 AZ Amsterdam, The Netherlands; elenaarbelo@secardiologia.es; 4 Pediatric Cardiology Unit, Hospital de Santa Cruz, Centro Hospitalar Lisboa Ocidental, 2790-134 Lisboa, Portugal; 5 Cardiovascular Diseases Research Group, Bellvitge Biomedical Research Institute (IDIBELL), 08908 Hospitalet de Llobregat, Spain; carles.diezlopez@gmail.com; 6 Advanced Heart Failure and Heart Transplant Unit, Department of Cardiology, Bellvitge University Hospital, 08908 Hospitalet de Llobregat, Spain; 7 Centro de Investigación Biomédica en Red, Enfermedades Cardiovasculares (CIBERCV), 28029 Madrid, Spain; 8 Arrhythmia Section, Cardiology Department, Hospital Clínic, Universitat de Barcelona, 08036 Barcelona, Spain; 9 Institut d’Investigació August Pi I Sunyer (IDIBAPS), 08036 Barcelona, Spain; 10 Department of Health Sciences, Section of Forensic Medical Sciences, University of Florence, Largo Brambilla 3, 50134 Florence, Italy; simone.grassi@unifi.it; 11 Department of Health Surveillance and Bioethics, Section of Legal Medicine, Fondazione Policlinico A. Gemelli IRCCS, Università Cattolica del Sacro Cuore, 00168 Rome, Italy; antonio.oliva@unicatt.it; 12 Medicine Department, School of Medicine, University of Cádiz, 11003 Cádiz, Spain; rocio.toro@uca.es; 13 Medical Science Department, School of Medicine, Universitat de Girona, 17003 Girona, Spain; 14 Institut d’Investigació Biomèdiques de Girona (IDIBGI), 17190 Salt, Spain

**Keywords:** sudden cardiac death, inherited arrhythmogenic syndromes, genetics, clinical interpretation, variants of unknown significance

## Abstract

**Background/Objectives:** Inherited arrhythmogenic syndromes comprise a heterogenic group of genetic entities that lead to malignant arrhythmias and sudden cardiac death. Genetic testing has become crucial to understand the disease etiology and allow for the early identification of relatives at risk; however, it requires an accurate interpretation of the data to achieve a clinically actionable outcome. This is particularly challenging for the large number of rare variants obtained by current high-throughput techniques, which are mostly classified as of unknown significance. **Methods:** In this work, we present a new algorithm for the genetic interpretation of the remaining rare variants in order to shed light on their potential clinical implications and reduce the burden of unknown significance. **Results:** Our study illustrates the potential utility of our individualized comprehensive stepwise analyses focused on the rare variants associated with IAS, which are currently classified as ambiguous, to further determine their trends towards pathogenicity or benign traits. **Conclusions:** We advocate for personalized disease-focused population frequency data and family segregation analyses for all rare variants that remain ambiguous to further clarify their role. The current ambiguity should not influence medical decisions, but a potential deleterious role would suggest a closer clinical follow-up and frequent genetic data review for a more personalized clinical approach.

## 1. Introduction

Inherited arrhythmogenic syndromes (IASs) are a group of rare genetic disorders that include cardiac channelopathies and cardiomyopathies. IASs are characterized by malignant arrhythmias that can lead to syncope and/or sudden cardiac death (SCD), which sometimes might be the first manifestation of the disease. This underscores the importance of early diagnosis and treatment. However, the variable clinical expressivity, incomplete penetrance, and overlapping phenotypes constitute a major challenge for their clinical management [1,2]. Genetic testing may help in the diagnosis of an IAS and the early identification and stratification of asymptomatic relatives, thereby enabling one to adopt a more personalized therapeutic approach and reduce the risk of lethal episodes [3].

The current guidelines include genetic analysis as a relevant tool in the diagnosis of IASs; however, the clinical yield of these studies is restricted to the identification of genetic variants classified as pathogenic or likely pathogenic [4,5,6]. In the context of IAS, the American College of Medical Genetics and Genomics and the Association for Molecular Pathology (ACMG/AMP) provide criteria for the accurate assessment of the variants classified as rare, contributing to an increase in the accuracy; however, they also imply some stringency [7]. This is extremely relevant when considering all of the IAS scenarios, where up to 50% of the missense variants might be currently classified as having uncertain significance (VUS) [8]. Moreover, the numbers of VUS tend to be higher in pathologies with low penetrance, due to a lack of data or inconsistencies in the available data, although some might turn out to be pathogenic after further comprehensive analyses [9]. Therefore, the overwhelming growing number of VUS driven by widespread genetic testing constitutes a real challenge and a need for deciphering to define their role in IASs [1,2,4,10].

Alternative systems for the clarification of VUSs have been suggested, but their utility in discriminating the clinical role of a VUS in an IAS has not yet been validated [11]. The classification of a genetic variant as a VUS impedes a useful clinical translation, leaving both families and clinicians facing uncertainty. In these cases, decision making should include only medical and family history for risk assessment and clinical management [1,2]. In the forensic field, IASs are highly suspected to be the most plausible cause of death due to the absence of evident anomalies, and a forensic conclusion often relies substantially on a molecular autopsy [12]. Finding a conclusive diagnosis at post mortem genetic analysis is also a public health priority, as it empowers clinicians with more information for a reliable risk assessment in relatives [13]. For these reasons, defining the significance of a VUS requires the collaboration and experience of a multidisciplinary group, helping to identify at-risk family members.

In recent years, an increasing need to clarify the role of VUS in IAS has facilitated the use of genetic data in clinical practice. A current approach to reduce ambiguity is the so-called re-analysis/re-classification/re-interpretation of VUSs, including newly available data [4]. Periodically updating VUSs may help to modify their ambiguous role in IASs and help to resolve the cases that remain to be clarified [14,15], possibly leading to significant benefits to the family and health care system [16]. Merging IAS-specific phenotype/clinical data within the variant interpretation process has become the standard of care in most expert institutions to reduce the ambiguity in identified VUSs [17,18]. Recently, the clinical use of VUS has been suggested only if a potential pathogenic role is strongly suspected [19] in a robust IAS-associated gene [20]. Along these lines, our group has recently incorporated an initial attempt to subclassify VUS categories in previously published studies, helping to obtain an approximate understanding of the definitive genetic role and subsequent clinical application in IASs [15,21,22,23,24]. We hypothesized that the use of our algorithm will help to further decipher the role of missense VUSs in IASs.

## 2. Materials and Methods

### 2.1. Study Cohort

Examples of index cases were included to show the application of criteria to unravel potentially suspicious deleterious missense variants currently classified as VUSs. Our retrospective study included 7 index cases with a definitive clinical diagnosis of IAS. The index case study included a comprehensive genetic analysis of all major and also minor genes currently associated with IAS (*ABCC9*, *ACTC1*, *ACTN2*, *AKAP9*, *ANK2*, *BAG3*, *CACNA1C*, *CACNA2D1*, *CACNB2*, *CASQ2*, *CAV3*, *CRYAB*, *CSRP3*, *DES*, *DMD*, *DSC2*, *DSG2*, *DSP*, *EMD*, *FBN1*, *FKTN*, *GLA*, *GPD1L*, *HCN4*, *JPH2*, *JUP*, *KCND3*, *KCNE1*, *KCNE2*, *KCNE3*, *KCNE5*, *KCNH2*, *KCNJ2*, *KCNJ5*, *KCNJ8*, *KCNQ1*, *LAMP2*, *LDB3*, *LMNA*, *MYBPC3*, *MYH6*, *MYH7*, *MYL2*, *MYL3*, *MYOZ2*, *MYPN*, *NEBL*, *NEXN*, *NOS1AP*, *PDLIM3*, *PKP2*, *PLN*, *PRKAG2*, *RANGRF*, *RBM20*, *RYR2*, *SCN1B*, *SCN2B*, *SCN4B*, *SCN5A*, *SGCD*, *SLMAP*, *SNTA1*, *TAZ*, *TCAP*, *TGFBR2*, *TGFB3*, *TMEM43*, *TMPO*, *TNNC1*, *TNNI3*, *TNNT2*, *TP63*, *TPM1*, *TRDN*, *TRPM4*, *TTN*, and *VCL*) [25] using a next-generation sequencing (NGS) custom-made resequencing panel approach, previously validated and already published [15]. The genetic analysis was approved by the ethics committee of Hospital Josep Trueta (Girona, Catalonia, Spain) according to the World Medical Association Declaration of Helsinki. Clinical and genetic data on all patients were anonymized and kept confidential (individual patient data will not be made available). Written informed consent was obtained from all patients before genetic analysis.

### 2.2. Data Sources

Five authors independently peer-reviewed all the available data (until August 2024) on each rare genetic variant; afterwards, the data were compared and verified, and a consensus was reached to avoid bias. All the investigators agreed on the final classification of all variants included. Different databases were consulted to obtain as much data as possible regarding variant–gene–IAS: the Genome Aggregation Database v4.1.0 (gnomAD) (https://gnomad.broadinstitute.org/), the Clinical Genome (ClinGen) (https://clinicalgenome.org/), VarSome (https://varsome.com/), the SCD-associated Variants Annotation Database (SVAD) (https://svad.mbc.nctu.edu.tw/), CardioClassifier (https://cardioclassifier.org/), InterVar (https://wintervar.wglab.org/), CardioVAI (https://cardiovai.engenome.com/), CardioBoost (https://cardiodb.org/cardioboost/), the Human Gene Mutation Database (HGMD) (http://hgmd.org), ClinVar (https://ncbi.nlm.nih.gov/clinvar/intro/), and the National Center for Biotechnology Information Single-nucleotide Polymorphism (SNP) Database (https://ncbi.nlm.nih.gov/SNP).

### 2.3. Interpretation

The genetic variants included in our study were previously classified following the ACMG/AMP recommendations (between 2016 and 2022). We verified that all of the rare variants remained classified as VUSs at the time of the analysis (August 2024), according to the ACMG/AMP recommendations [7]. Firstly, it should be mentioned that the VarSome database has included an approximate score of a VUS with a deleterious or benign role, but this role is suggested if the VUS score using ACMG/AMP items is near to the likely benign (LB) or likely pathogenic (LP) category, respectively. This is particularly challenging for missense variants, which is why we have developed a more precise algorithm specifically tailored to address them. The first key point in our approach is the use of a definite association gene–IAS [1,2]. In addition, the reported pattern of inheritance is also crucial to clarify the potential role of a missense VUS. Our approach is focused on clarifying the role of rare missense variants currently classified as VUSs according to the available data and the ACMG/AMP recommendations, not to report a novel gene associated with IAS. Recent updates on the ACMG/AMP recommendations have been used, such as the PM2 (moderate evidence of pathogenicity) item, which was considered fulfilled if the minor allele frequency (MAF) in the relevant population databases was ≤0.01% [26]. Other recent updates according to gene-specific [27] and disease-specific associations have also been considered [17,18]. All IASs are rare diseases (prevalence less than 1/2000, threshold MAF < 0.05%), except HCM (1/500, threshold MAF < 0.2%). The patient’s ethnic background should also be included to obtain a personalized genetic profile, as well as the threshold MAF [6], as occurs, for example, in Brugada syndrome (BrS) (increased prevalence in the Asiatic population) [28]. Therefore, each IAS has a different threshold following the reported prevalence, and it should be considered in each analyzed variant, in addition to the patient’s ethnicity. In addition, all the reported deleterious variants currently classified as definitively pathogenic in IASs following ACMG/AMP recommendations are very rare (MAF < 0.005%) [29]. Therefore, the frequency of a deleterious rare variant never exceeds the population prevalence of the specific disease. We have used this MAF percentage in our algorithm as the most restrictive item for rare variants with a possible deleterious role to discern them from a rare variant too common to be causative for IAS. Other MAFs with no deleterious role vary depending on the population prevalence of each disease (Table 1).

**Figure 1:**
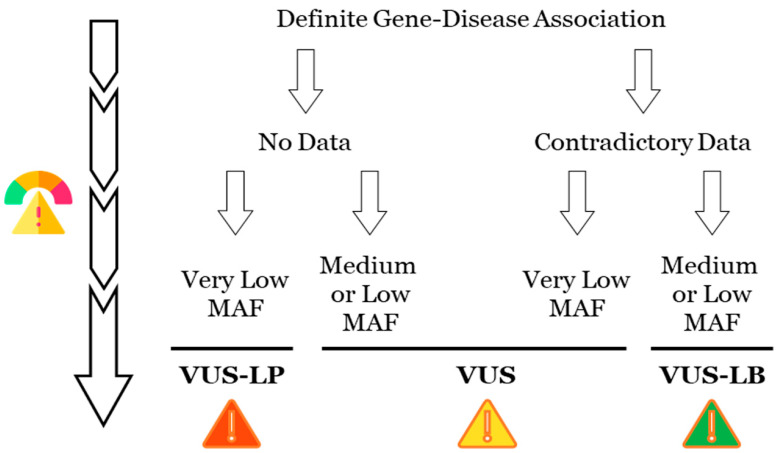
Diagram used in the interpretation of VUS. MAF: Minor allele frequency, VUS: variant of uncertain/unknown significance, VUS-LB: variant of uncertain/unknown significance—likely benign, VUS-LP: variant of uncertain/unknown significance—likely pathogenic.

It is important to consider that, due to the large number of cases currently included in the population databases, a novel variant in a definite disease-associated gene (with no available MAF) is considered as highly suspicious of playing a deleterious role (Table 1). Finally, the labelling of a variant as a VUS may be due to a lack of data or incongruences in the available data [10]. From our viewpoint, the lack of data implies a higher risk of a deleterious role in contrast to the contradictory available data. All these items should be considered to perform a comprehensive analysis of a missense variant classified as a VUS, as well as with a highly potential deleterious role in IAS (Figure 1). Thus, the application of our algorithm follows the sequential pattern indicated in Figure 1. First, we definitively associate the gene with the pathology. The second step would be the non-existence of information or available but contradictory data regarding the variant. Finally, the population frequency would allow us to achieve a more specific classification within this ambiguity. To this last point, and based on the frequency of each pathology, even considering the patient’s ethnicity, the criteria established in Table 1 would be applied. This would achieve a medium, low, or very low frequency. Recently, our group has published this approach focused on DCM [24].

## 3. Results

### 3.1. Cohort

Our study included the experience of more than 100 cases in which we applied this proposed approach, as recently published [22,23,24]. Now, we explain the algorithm in detail using examples of real cases, each one diagnosed as an IAS, as an example for each disease. The patients were diagnosed between 2016 and 2022 according to the clinical guidelines available at that time. The clinical data were updated for each case, and no changes in diagnosis were observed following the current guidelines update. Genetic analysis, including all genes associated with the diagnosed disease in each family (please, see the methods section) did not identify pathogenic (P) or LP variants in any of the genes currently associated with the definite diagnosed IAS. At least one rare missense variant classified as a VUS was identified in each case. These variants were classified at the moment of genetic analysis following the ACMG/AMP recommendations. Remarkably, the updated review of the clinical significance showed no changes in any of the missense VUSs; these retained an ambiguous role in IAS following the ACMG/AMP recommendations, leaving all cases without a conclusive genetic diagnosis (Table 2).

### 3.2. SCN5A_p.Ile768Val (Brugada Syndrome)

This example is a case where the patient was diagnosed with BrS due to spontaneous Brugada pattern type 1 on the electrocardiogram (ECG) after medical work revision (Shanghai score: 3.5 points). No syncope or clinical symptoms have been reported in our patient to date. The genetic analysis identified the missense rare variant p.(Ile768Val) in the *SCN5A* gene (HGNC: 10593). The amino acid was isoleucine (Ile, I)—aliphatic, essential—changes to valine (Val, V)—aliphatic, essential. This variant, not previously reported and without available data, was previously classified as a VUS following the ACMG/AMP recommendations. Now, after updating the available data and consulting the predictive databases, it remains classified as a VUS according to the ACMG/AMP recommendations. This classification is due to the lack of sufficient evidence to determine its significance in the context of the associated disease. Our algorithm suggests a potential deleterious role; therefore, it should be subclassified as a VUS-LP. Taking all the data into account, the genetic carriers should undergo clinical assessment, and preventive measures should be adopted (if any symptom is observed), always following the current clinical guidelines on IASs. If the patient is asymptomatic, we suggest a close clinical follow-up (Table 2).

### 3.3. KCNH2_p.Gln81Pro (Long QT Syndrome)

This example is a case diagnosed with LQTS (QTc: 485ms, Schwartz score: 4 points). This patient has not complained of syncope or any clinical symptoms to date. Pharmacological treatment with betablockers was recommended. The genetic analysis identified the missense rare variant p.(Gln81Pro) in the *KCNH2* gene (HGNC: 6251). The amino acid was glutamine (Gln, Q)—amidic, essential—changes to proline (Pro, P)—aliphatic, nonessential. This variant was previously classified as a VUS following the ACMG/AMP recommendations. No data were available up to this point, and according to the consulted databases, it remains classified as a VUS following the ACMG/AMP recommendations due to the lack of available data. Our algorithm suggests a potential deleterious role; therefore, it should be subclassified as a VUS-LP. Taking all the data into account, genetic carriers should be clinically assessed and preventive measures adopted (if any symptom is observed), always following the current clinical guidelines on IAS. If a patient is asymptomatic, we advise regular clinical follow-up (Table 2).

### 3.4. KCNH2_p.Arg1135His (Short QT Syndrome)

Our patient showed a borderline SQTS (QTc: 365ms, SQTS score: 3 points). Neither syncope or clinical symptoms have been reported to date, despite the patient being clinically assessed due to palpitations and chest pain. The genetic analysis identified the missense rare variant p.(Arg1135His) in the *KCNH2* gene (HGNC: 6251). The amino acid was arginine (Arg, R)—basic, nonessential—changes to histidine (His, H)—basic, essential. This variant (rs199473547) shows a very low population frequency (MAF:0.0003%), and it was previously reported in an individual with a short QT interval and BrS overlapping phenotype (showing a baseline ECG with Brugada type 1 pattern) [30]. Functional studies showed that this change caused a gain of function in the *KCNH2* gene, which could be related to the phenotype [31]. Therefore, with this evidence, it was classified with an ambiguous role in ClinVar. It was previously classified as a VUS following the ACMG/AMP recommendations. Now, after consulting the databases, we classified it as a VUS following the current ACMG/AMP recommendations despite the available data. Our algorithm suggests a modified role as a VUS-LP, especially due to the very low frequency. Taking all the data into account, the genetic carriers should be clinically assessed, but no measures should be adopted that include this genetic variant. It should be noted that the segregation of the variant within these families plays an important role in clarifying its involvement in causing the disease and in facilitating the establishment of a definitive diagnosis (Table 2).

### 3.5. RyR2_p.Val4768Ile (Catecholaminergic Polymorphic Ventricular Tachycardia)

This example is a patient diagnosed with CPVT after an exercise test (CPVT score: 4.5 points). The genetic analysis identified the missense rare variant p.(Val4768Ile) in the *RyR2* gene (HGNC: 10484). The amino acid was valine (Val, V)—aliphatic, essential— changes to isoleucine (Ile, I)—aliphatic, essential. It was previously reported (rs775534249) with a very low population frequency (MAF:0.002%) and classified as having an ambiguous role in ClinVar. Therefore, it was classified as a VUS following the current ACMG/AMP recommendations. With this update and through consulting the databases, there was a consensus to classify the variant as a VUS, also according to the ACMG/AMP recommendations, but our algorithm suggests a tendency toward an ambiguous role. Thus, it should remain as a VUS. Taking all the data into account, the genetic carriers should be clinically assessed, but no measures should be adopted that include this genetic variant. Only clinical data (if any) should be considered in management. It should be noted that the segregation of the variant in these families plays an important role in clarifying its role in the causation of the disease and in facilitating the establishment of a definitive diagnosis (Table 2).

### 3.6. MYH7_p.Lys1459Asn (Hypertrophic Cardiomyopathy)

This example is a patient with a definite diagnosis of HCM. The genetic analysis identified the missense rare variant p.(Lys1459Asn) in the *MYH7* gene (HGNC: 7577). The amino acid was lysine (Lys, K)—basic, essential—changes to asparagine (Asn, N)—amidic, nonessential. This variant was previously identified in multiple cases of HCM and DCM, among others, and in asymptomatic (rs201307101), and it was previously classified as a VUS following the ACMG/AMP recommendations. Now, the available data show controversial results in different databases, suggesting a current classification as an LB or VUS, always following the current ACMG/AMP recommendations. The frequency of 0.029% suggests a VUS-LB role following our algorithm. Taking all the data into account, at least first-degree relatives should be clinically assessed, but no measures should be adopted for this genetic variant. It is worth noting that the segregation of the variant in these families plays a significant role in either confirming a potential deleterious role or excluding the variant as the causative factor. This last role is critically important in reducing the anxiety of the genetic carriers and increasing the accuracy of genetic interpretation (Table 2).

### 3.7. TTN_p.Pro877Thr (Dilated Cardiomyopathy)

This example is a definite diagnosis of DCM. The genetic analysis identified the missense rare variant p.(Pro877Thr) in the *TTN* gene (HGNC: 12403). The amino acid was proline (Pro, P)—aliphatic, nonessential—change to threonine (Thr, T)—hydroxylic, essential. This variant was previously identified only in a private laboratory analysis (rs751640052). It was previously classified as a VUS following the ACMG/AMP recommendations. Now, the MAF is extremely low (0.0006%), and no additional data are available to date. Therefore, consulting the databases, we suggest that it should remain classified as a VUS following the current ACMG/AMP recommendations. However, our algorithm suggests a potential deleterious role, and it should be considered a VUS-LP. Taking all the data into account, the genetic carriers should be clinically assessed, and preventive measures should be adopted (if any symptom is observed), always following the current clinical guidelines. If a patient is asymptomatic, we suggest a close clinical follow-up due to the harboring of a deleterious genetic variant, which increases the risk of developing cardiomyopathy in comparison with non-carriers (Table 2).

### 3.8. DSG2_p.Gly1089Asp (Arrhythmogenic Cardiomyopathy)

This example was a female index case with a definite diagnosis of ACM, according to the Task Force Criteria (one major and two minor). The genetic analysis identified the missense rare variant p.(Gly1089Asp) in *DSG2* (HGNC: 3049). The amino acid was glycine (Gly, G)—aliphatic, nonessential—changes to aspartic acid (Asp, D)—sulfur-containing, nonessential. This variant was previously identified (rs200264407) and classified as a VUS following the ACMG/AMP recommendations. Now, the available data show controversial results, and the consulted databases suggest a classification as an LB or VUS, according to the current ACMG/AMP recommendations. The frequency of 0.011% suggests a VUS-LB role following our algorithm. This fact highly suggests that *DSG2*_p.(Gly1089Asp) is not a cause of ACM, although we could not rule out its implication in ACM. Taking all the data into account, the genetic carriers should be clinically assessed, but no measures should be adopted for this genetic variant. It should be noted that the segregation of the variant in these families plays an important role in clarifying a potential deleterious role or removing the variant as causative. This last role is of key importance in reducing the anxiety of the genetic carriers and in increasing the accuracy of the genetic interpretation (Table 2).

## 4. Discussion

Genetic testing is recommended in families with a confirmed or suspected diagnosis of IAS, and an accurate interpretation is crucial to discern the specific role that a genetic variant play, allowing for useful translation in clinical settings [1,2]. An increased risk of developing cardiomyopathy when harboring a deleterious genetic variant has been reported, highlighting the crucial role of a suitable genetic interpretation [32]. Focusing on DCM, patients who harbor a rare variant classified as P/LP showed an increased risk of suffering the most severe phenotypes [33]. This fact reinforces the use of diagnostic gene panels limited to definite IAS-associated genes, as proposed in our algorithm.

Currently, the genetic diagnosis of IAS follows the ACMG/AMP recommendations, which include a large number of items that allow for a consistent classification [7]. In IASs, this “one-size-fits-all” approach increases the VUS burden and fails to incorporate the clinical phenotypes into the variant classification. The clinical data of the patient as well as those of their relatives should be carefully analyzed before making a genetic diagnosis [8]. This genetic report is especially challenging for missense VUSs, whose functional consequences are harder to predict [34]. We have focused on this kind of genetic alteration, classified as VUS in accordance with the updated ACMG/AMP recommendations. Combining the IAS-specific phenotype and clinical data helped to reduce the ambiguity of the identification [17,18] of *RyR2* in CPVT cases and *KCNQ1* in LQTS cases. We consider a definitive clinical diagnosis to be a crucial factor, which is why we have included it in our approach as the threshold. We have focused on definite VUS without role modifications after incorporating a standardized assessment of the phenotypic strength into the variant classification, in accordance with the ACMG/AMP recommendations. In the context of IAS, variants classified as VUSs should not be used in clinical management but cannot be omitted as a potential cause of the disease, highlighting a periodic revision until a definite role in IAS is obtained [14]. In 2020, a study focused on Loeys–Dietz syndrome suggested that some VUSs with a potential deleterious role (VUS-LPs) could be assumed to be de novo according to the family history [35]. Recently, the clinical use of VUSs has been suggested as a “hot VUS”, with a high suspicion of pathogenicity, depending on the strength of the phenotype, combined with the chance of finding a VUS in that specific gene [19]. A recent study has suggested that VUSs can be associated with an increased risk of cardiac mortality and heart failure hospitalization, especially VUSs located in definite IAS-associated genes [20]. In concordance, we recommend that patients with a predicted VUS-LP receive close follow-up despite being asymptomatic. However, neither of the abovementioned studies produced an algorithm to obtain this suspected deleterious classification of VUS as we define in our study. Therefore, in addition to the rules currently used in genetic interpretation, we propose a more specific approach focused on increased sensitivity for VUSs, showing a tendency towards a benign (VUS-LB) or deleterious (VUS-LP) role in IASs. With the aim of advancing the personalized diagnosis of IAS, a subclassification of VUS was previously suggested in studies of our group [15,21,22,23,24], helping mainly to discern benign background “genetic noise”, although it still needs to be validated before being implemented in clinical practice.

**Figure 2:**
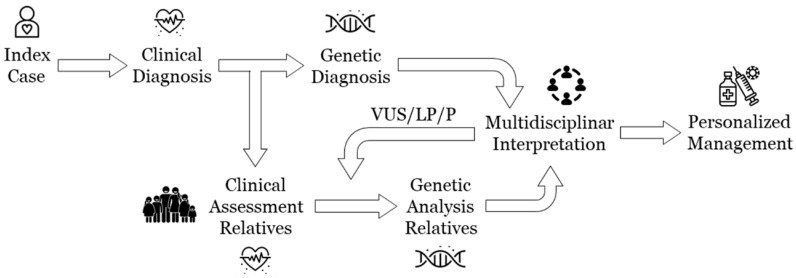
The clinical and genetic algorithm used in the interpretation of VUSs. LP: Likely pathogenic, P: pathogenic, VUS: variant of uncertain/unknown significance.

The use of NGS technology allows for wide genetic screening, identifying a large number of rare variants despite most of them being located in genes not definitively associated with IAS, named genes of uncertain significance (GUSs) [4,5]. If there is no definite association with an IAS, a variant should always be classified as a VUS and only be reclassified when a conclusive association between the gene and an IAS is identified. For diagnostic purposes, only a well-established gene–IAS association should be utilized, and disease-specific phenotypes significantly increase the accuracy of a genetic classification [36]. In addition, personalized use of the current global prevalence of an IAS compared with the available MAF data for VUS should be considered [34]. From our point of view, the frequency of rare variants in the global population should be one of the main tools used to discern a potentially deleterious variant from the genetic background. We have used a stringent population frequency threshold based on the current IAS prevalence, allowing us to identify interpretable variants with high confidence. Due to continuous improvements in understanding the genetic background of IAS, it is imperative to regularly update the available MAF periodically. This can lead to a definitive alteration of a prior uncertain classification, often resulting in a reduced deleterious impact [14]. Finally, from our perspective, the genetic segregation of VUSs in relatives is crucial to clarify their role in IAS (Figure 2). Current clinical guidelines recommend offering genetic testing to family members when a definite deleterious variant is identified [1,2]. However, a key point that helps to clarify the deleterious role of a VUS is familial segregation, even though incomplete penetrance and variable expressivity generally impede achieving a conclusive result [4]. Our experience in the field of IAS supports a family segregation of all VUSs identified; in most cases, cascade testing may not clarify a pathogenic role, but it does facilitate a reduction in a potential deleterious role. Obtaining this decreased deleterious role is important to note, as these VUS can be disregarded as the main cause of disease in each patient, reducing the genetic ambiguity as well as the anxiety in a genetic carrier. It is important to note that a modification in the genetic classification does not imply a different clinical diagnosis, nor does it imply that the patient does not suffer from a previously diagnosed IAS. In concordance with recent studies, we agree that a group of experts in cardiogenetics, including at least clinical geneticists and genetic counsellors, should explain to families what reclassification involves to avoid misunderstanding [37], considering the complexity of understanding VUSs [38,39].

Our interpretation of missense VUSs in IASs has some limitations that should be mentioned. Firstly, the patients included in our cohort might carry pathogenic variants in genes not yet associated with the diagnosed IASs. Secondly, the use of available MAF helps to discern a high percentage of the genetic background that is not detrimental in IAS, as demonstrated by our results. However, we cannot rule out the possibility that future population studies may modify some of the MAFs in VUSs. This point is an important limitation concerning lack of ethnicity and population-specific data. Concerning family screening, at least three generations should be included to obtain an informative segregation following the current guidelines. Our algorithm does not include this point due to the fact that, in many of the published cases, no family segregation is available, so our algorithm may help to discern a conclusive role when no relatives are available (although we highlight the crucial role of family segregation, if available). In the same way, variants following different patterns of inheritance should be exhaustively analyzed due to potential modifications in interpretation. In addition, a lack of available functional data impedes more accurate interpretation, causing ambiguous roles to remain in IAS. It is also important to mention that our study exclusively focuses on missense VUSs, which give the most ambiguity in IASs. The “radical” variants associated with IASs (nonsense, indels, frameshift, and copy number variants) have not been analyzed following our algorithm; hence, further studies should be carried out to validate our approach in these types of “radical” variants. In addition, it still remains to be analyzed whether other cardiac diseases of genetic origin could benefit from the improvements proposed in our study. Finally, our subclassification could be subject to inherent intra- and inter-laboratory differences in data interpretation [40]; thus, it should also be tested in other centers and in large cohorts to corroborate our results on IAS.

## 5. Conclusions

The ambiguous role of variants previously labelled as VUSs precludes their potential usefulness in clinical practice. Reviewing and re-interpreting these variants in their potentially benign or pathogenic role may help to obtain actionable data. We demonstrate the potential value of a new algorithm that is straightforward and swift to implement. Our algorithm incorporates a confirmed clinical diagnosis, the analysis of contradictory or unavailable data for VUSs, and the utilization of population frequencies according to the prevalence of IASs. The use of our approach, as well as family segregation of missense VUS, helps to clarify their role in IASs. Although family segregation of VUSs is currently a topic of debate, it may help to clarify a definite cause of the disease, at least reducing the ambiguity and alleviating anxiety in genetic carriers. We recommend that genetic carriers of missense VUS-LPs receive a close clinical follow-up due to the high likelihood of pathogenicity.

## Figures and Tables

**Table 1:** Frequencies used in the interpretation of VUSs. ACM: Arrhythmogenic cardiomyopathy; BrS: Brugada syndrome; CPVT: catecholaminergic polymorphic ventricular tachycardia; DCM: dilated cardiomyopathy; HCM: hypertrophic cardiomyopathy; IAS: inherited arrhythmogenic syndrome; LQTS: long QT syndrome; MAF: minor allele frequency; SQTS: short QT syndrome; VUS: variant of uncertain/unknown significance.

IAS		MAF		
	Prevalence (MAF)	Medium	Low	Very Low
BrS	1/2000 (0.05%)	>0.05%	≤0.05% ≥0.005%	<0.005% or No MAF
LQTS	1/2000 (0.05%)	>0.05%	≤0.05% ≥0.005%	<0.005% or No MAF
SQT	1/20000 (0.005%)	>0.005%	≥0.005%	<0.005% or No MAF
CPVT	1/10000 (0.01%)	>0.01%	≤0.01% ≥0.005%	<0.005% or No MAF
HCM	1/500 (0.2%)	>0.2%	≤0.2% ≥0.005%	<0.005% or No MAF
DCM	1/2500 (0.04%)	>0.04%	≤0.04% ≥0.005%	<0.005% or No MAF
ACM	1/5000 (0.02%)	>0.02%	≤0.02% ≥0.005%	<0.005% or No MAF

**Table 2:** Data of VUSs following the ACMG/AMP recommendations (updated August 2024). ACM: Arrhythmogenic cardiomyopathy; BrS: Brugada syndrome; CPVT: catecholaminergic polymorphic ventricular tachycardia; DCM: dilated cardiomyopathy; HCM: hypertrophic cardiomyopathy; IAS: inherited arrhythmogenic syndrome; LQTS: long QT syndrome; NA: not available; SQTS: short QT syndrome; VUS: variant of uncertain/unknown significance; VUS-LB: variant of uncertain/unknown significance—likely benign; VUS-LP: variant of uncertain/unknown significance—likely pathogenic.

Disease	Gene	Nucleotide	Protein	dbSNP/ClinVar	GnomAD (%)	Data	Suggested Role
BrS	*SCN5A*	c.2302A>G	p.(Ile768Val)	NA/VUS	NA	NA	VUS-LP
LQTS	*KCNH2*	c.242A>C	p.(Gln81Pro)	NA/NA	NA	NA	VUS-LP
SQTS	*KCNH2*	c.3404G>A	p.(Arg1135His)	rs199473547/VUS	5/1568264 (0.0003)	NA	VUS-LP
CPVT	*RyR2*	c.14302G>A	p.(Val4768Ile)	rs775534249/VUS	34/1613388 (0.002)	C	VUS
HCM	*MYH7*	c.4377G>T	p.(Lys1459Asn)	rs201307101/LB	739/1614024 (0.04)	C	VUS-LB
DCM	*TTN*	c.2629C>A	p.(Pro877Thr)	rs751640052/VUS	1/152210 (0.0006)	NA	VUS-LP
ACM	*DSG2*	c.3266G>A	p.(Gly1089Asp)	rs200264407/LB	115/1614020 (0.007)	C	VUS-LB

## Data Availability

All the data that support the findings of this study are in the manuscript.
